# Feature binding and error commission

**DOI:** 10.3758/s13414-025-03164-w

**Published:** 2026-01-13

**Authors:** Anna Foerster, Svante Linz, Birte Moeller, Maria Nemeth, Christian Frings, Roland Pfister

**Affiliations:** Trier University, 54286 Trier, Germany

**Keywords:** Action control, Binding, Retrieval, Error processing

## Abstract

Perceptual and action representations consist of multiple independent features such as color and location of an encountered stimulus, or effector and direction of a performed action. Performing an action further establishes bindings between perceptual and action features, so that reencountering one feature retrieves all bound features. When errors are committed, both erroneous and correct responses are usually strongly represented. In Experiment 1, we investigated the binding between erroneous responses and their effects for different types of errors, with the goal of replicating and generalizing a previous single finding. In Experiment 2, we investigated whether perceptual features bind to correct or erroneous responses depending on whether they appear before or after response execution. These bindings had so far been studied separately. Participants categorized letters via key-press responses, and an irrelevant sound was played after their response (Exp. 1 and 2) or before (Exp. 2 only). Then the same or another sound was played, signaling participants to spontaneously choose a response. After an error in the letter task, participants chose the previous erroneous response more often when the sound was repeated than when it was changed. Surprisingly, neither the error type nor the timing of the sound relative to the response modulated this preference. Thus, the data unanimously support binding and retrieval between perceptual features and erroneous responses. Whether and how binding and retrieval also emerge for the nonexecuted correct response, however, seems to depend on contextual factors and might not be as ubiquitous as has been suggested before.

## Introduction

Even if agents know the necessary steps to reach a goal, they still can err in implementing these steps. Luckily, errors are dealt with swiftly. For one, erroneous actions are terminated earlier than correct actions, suggesting that their execution can be cancelled on the fly (Foerster et al., 2022a, 2022b; Hochman et al., 2017). Second, correction responses occur shortly after the error, suggesting that they are already selected during the commission of errors (Crump & Logan, 2013; Fiehler et al., 2005; Rabbitt, 1966, 2002). Even if correction responses are not executed, sub-threshold motor activity of the correct response is measurable in the context of error commission (Beatty et al., 2021; Foerster et al., in press). That is, both an executed erroneous response and the not executed correct counterpart are active when agents err. The two responses are also active during following responses and affect performance (Foerster et al., in press).

Recently, we have been exploring whether and how these two active responses of an error are integrated with perceptual aspects of the situation in which the error occurs (Foerster et al., 2021, 2023, 2024; Foerster et al., 2022a; Parmar et al., 2022). The integration of perception and action is theoretically grounded in contemporary action control frameworks (e.g., Theory of Event Coding; Hommel et al., 2001, Binding and retrieval in Action Control; Beste et al., 2023; Frings et al., 2020). These frameworks propose that attributes—so-called features—of stimuli, responses and effects of these responses are linked to each other (Frings et al., 2024). For example, a red light requires pulling the brake to stop a bicycle. Stimulus features like red, response features like pulling a lever with the right hand, and effect features like the bike coming to a halt, are active in this situation. For correct actions, decades of research provide evidence that bindings exist between features of stimuli and responses and of responses and effects (e.g., Dutzi & Hommel, 2009; Hommel, 1998; Moeller et al., 2016, 2019).^1^ That is, performing an action leads to response features being bound to the preceding stimulus features or the following effect features. When a stimulus or effect feature becomes reactivated again afterward, it retrieves the bound response feature. For example, binding a red color to a right hand pull should retrieve the latter response when seeing this color again. The retrieval of a response feature facilitates the execution of the same response but hampers the execution of other responses. Binding and retrieval processes therefore create short-cuts to recent actions. They are empirically assessed either via performance data (response times and error rates) or via choice data. For performance data, binding effects emerge from the interplay of stimulus/effect sequence and response sequences in sequential analyses of choice response tasks (Frings et al., 2024). For choice data, binding effects materialize as the impact of stimulus/effect sequence on immediately following response choices.

How binding operates for erroneous actions, by contrast, has only recently been targeted by empirical research. This research tested whether there is any binding at all (what had been assumed previously; Hommel, 2005), and what features are eventually bound. Interestingly, the evidence points to binding and retrieval of the correct and the erroneous response alike. Multiple experiments indicate that the not executed correct response can be bound to a stimulus. First, binding effects emerged between features of *task-relevant* stimuli and correct responses (Exp. 1 in Foerster et al., 2022a; Foerster et al., 2023). These bindings mirror the instructed task rule about the assignment of stimuli to responses and steer agents back toward successful action control. Second, binding effects also emerged between features of *irrelevant* stimuli and correct responses (Foerster et al., 2021; Parmar et al., 2022). Third, for the executed erroneous response, one published experiment provided evidence for binding of response features with features of *irrelevant* effects (Exp. 2 in Foerster et al., 2022a). Stimuli and effects in these latter experiments were irrelevant because the correct response could not be inferred from their identity. In other words, there were neither instructed rules nor regularities between features of stimuli/effects and responses, allowing for an investigation of short-term bindings in the absence of long-term memory traces.

**Fig. 1:**
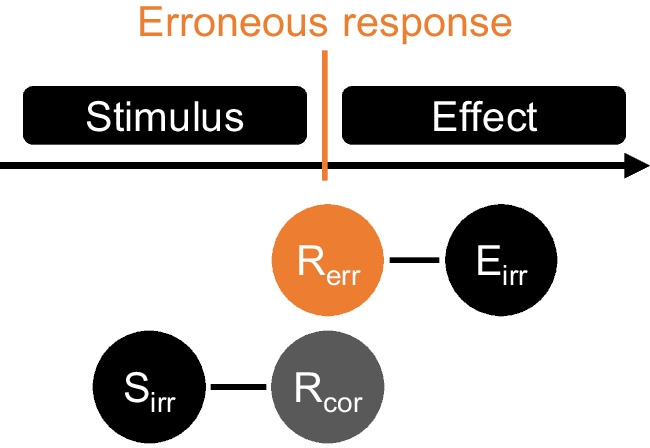
Depiction of the hypothesized bindings. *Note.* For erroneous episodes, there is evidence for binding between features of an irrelevant stimulus (S_irr_) and a not executed correct response (R_cor_) and between features of an executed erroneous response and an irrelevant effect (E_irr_). Experiment 1 assessed the replicability and generalizability of R_err_–E_irr_ bindings for different error types. Experiment 2 scrutinized whether irrelevant perceptual features enter bindings with the correct response whenever they appear as stimulus (i.e., before the response) but with the erroneous response whenever they appear as effect (i.e., after the response)

The first aim of the current research was to corroborate the previous finding that binding and retrieval processes emerge for erroneous responses and their irrelevant effects. This was implemented in Experiment 1 (see Fig. 1). At the same time, we explored the generalizability of these processes across different types of errors. The second aim was to investigate whether response execution serves as a switch that determines whether features of perceptual events are bound to either the (not executed) correct response or to the erroneous response. This was implemented in Experiment 2. That is, we directly assessed whether irrelevant perceptual features are bound to the not executed correct or the executed erroneous response, depending on whether they are presented before or after response execution.

## Experiment 1

Bindings between erroneous responses and their effects have previously been studied in a design where each trial featured a prime and a probe segment (Exp. 2 in Foerster et al., 2022a). In the prime, participants categorized one out of eight target numbers at a time as odd or even. The response triggered an effect sound that was randomly low-pitched or high-pitched. We assumed that binding would take place between the response and the sound in the prime. As soon as one of the sounds played in the probe, participants had to choose one of the response keys spontaneously. Participants used the same two response keys in the prime and the probe. We hypothesized that sound repetitions from prime to probe would trigger the retrieval of the response that was bound to this sound in the prime. Retrieval of a response should increase the frequency of choosing it instead of the other response in the probe. In trials with an erroneous response in the prime, the erroneous prime response was more frequently chosen in the probe when the sound repeated than changed. This effect of the sound sequence on response choices in the probe points to retrieval of bindings between the erroneous response and the sound.

In the former design, the commission of errors could not be traced back to a systematic activation of wrong information via stimulus processing. Instead, we provoked wrong key presses in the prime in general by implementing a short response deadline. Further, we presented irrelevant letters that surrounded the centrally presented target letter. However, these letters only increased visual noise, but they were not assigned to the response keys. That is, errors probably emerged whenever a spontaneous activation of an incorrect response surpassed the response threshold more quickly than the rule-based activation of the correct response did.

In the current study, we introduced an identifiable source of error commission based on erroneous response activation from an irrelevant stimulus. We implemented a flanker task where a target letter was presented amongst a grid of irrelevant (flanking) letters. Target and irrelevant letters were selected from the same stimulus set (i.e., six letters) that we assigned to three responses (adapted from Maier et al., 2008; see also Eriksen & Eriksen, 1974). Participants had to respond to the identity of the target letter and ignore the irrelevant letters. The interesting condition is when two different letters that are assigned to incongruent responses are presented as target and irrelevant stimuli. In this case, the error either matches the response assigned 1) to the irrelevant letter (flanker error) or 2) neither to the target nor to the irrelevant letter (nonflanker error).

While both error types could have various causes, only flanker errors can be caused specifically by a failure to selectively attend the target stimulus while ignoring the surrounding irrelevant stimuli. Previous research investigated the consequences of this selection failure for error processing. For one, little attention on the relevant stimulus supposedly reduces correct response activation after flanker errors, hampering error detection (Maier et al., 2008). In line with this assumption, participants signaled flanker errors less frequently than nonflanker errors. Second, selection failures that cause flanker errors should result in a readjustment of attention toward greater selectivity afterward (Maier et al., 2011). This assumption was supported as incongruent flanker trials provoked more errors compared to neutral trials only following nonflanker errors but not following flanker errors.

Two competing hypotheses can be derived from how the selection failure in flanker errors could affect binding and retrieval effects compared to nonflanker errors. On the one hand, bindings could be stronger if the executed and bound erroneous response faces less competition from the correct response, and if cognitive processing is therefore less engaged in the detection of flanker errors. On the other hand, increased selectivity after flanker errors might hamper automatic retrieval of bindings. As binding and retrieval are measured via the same interaction between response and effect sequence, this interaction could therefore be either stronger or weaker for flanker than nonflanker errors.

We assessed retrieval of bindings between prime responses and their effects in choice frequencies of probe responses by comparing distinct prime–probe sequences—namely, the orthogonal combination of the sound sequence (repetition vs. change) and the repeated response. After responding correctly in the prime, participants could choose this prime response also in the probe (termed *correct response of a correct prime* in the following) or the other two (not executed) neutral responses. After responding erroneously in the prime, the probe response could either match the correct (not executed) prime response (i.e., *correct response of an erroneous prime*), or the erroneous (executed) prime response (i.e., *erroneous response of an erroneous prime*), or the (not executed) neutral response. A decisive advantage of having three response options is therefore that binding of the erroneous and the correct response can be assessed at the same time for errors. In the former two-choice paradigm without a neutral response option, erroneous and correct responses could only be pitted against each other (Exp. 2 in Foerster et al., 2022a). In theory, both bindings might have been present in this design. However, the empirical effects may have only captured stronger or more prevalent bindings of the erroneous response, thereby obscuring binding of the correct response.

Binding of an executed (correct or erroneous) response or a not executed correct response to the sound in the prime would be reflected in an increased likelihood of repeating the respective response when the sound repeats compared to when it changes. We expected sound repetitions relative to changes to increase the repetition frequency for the correct response of a correct prime in the probe. We further expected sound repetitions relative to changes to increase the repetition frequency for the erroneous response of an erroneous prime. The strength of this binding effect could differ between flanker and nonflanker errors. Lastly, the correct response of an erroneous prime should be repeated more frequently after a nonflanker error than after a flanker error if the former error type comes with stronger correct response activation.

### Method

#### Participants

The effect size for binding following erroneous primes in response choices was *d*_*z*_ = 0.32 in our preceding study (Exp. 2 in Foerster et al., 2022a). Effect sizes across correct and erroneous primes were larger. A sample of 79 participants has a power of 80% to detect the small effect size (*d*_*z*_ = 0.32) in a two-tailed test with α = 5% (computed with the *power.t.test* function in R Version 4.0.3). We decided to collect a sample of 78 analyzable participants for counterbalancing, which still provides about 80% power.

Five participants aborted the study prematurely and had to be replaced. Ninety-nine participants provided full datasets of which we had to exclude 21 participants as per our preregistered criteria. Of the remaining 78 participants, 54 reported to be female and 24 male; 72 right-handed, five left-handed, one ambidextrous. The mean age was 24.5 years (*SD* = 4.57 years).

We planned to collect pilot samples of up to 8 participants each. Our goal was to exclude two participants (25%) at a maximum based on predetermined criteria (see *Data Treatment*). If a pilot sample did not fulfil this goal, we would adapt the paradigm and invite a new sample to test the novel design. If a pilot sample fulfilled the goal after 8 participants, we would fill up the sample according to the power analysis above. Four pilots failed because we had to exclude more than 25% of the participants. We excluded 5/6 participants in Pilot 1, 4/8 participants in Pilot 2, 5/8 participants in Pilot 3 and 6/8 participants in Pilot 4. In the fourth pilot sample, however, 7/8 (>75%) participants could be included in the analysis when adapting one of the exclusion criteria, namely when lowering the criterion for minimum cell observations to five (instead of 10 as preregistered). We decided to continue with this fourth version and the adapted exclusion criterion after piloting and filled up the sample according to the power analysis above. We updated the existing preregistration to include this change. We summarized methodological differences between the pilots in Appendix A.

#### Apparatus and stimuli

Participants conducted the experiment on computers. We used the letters *B*, *K*, *P*, *R*, *M*, and *V* as target and irrelevant stimuli*.* We always paired the letters *B* with *K*, *P* with *R*, and *M* with *V*, but we counterbalanced the assignment of these pairs to three keys across participants. Participants responded with the index, middle and ring finger of their dominant hand on the adjacent arrow keys that point left, down and right on a QWERTZ keyboard. They wore headphones to hear the prime and probe sounds (i.e., 400 Hz and 800 Hz) as well as catch sounds (600 Hz).

We presented visual stimuli in white font and accuracy feedback in red or green font against a black background. The fixation cross always appeared centrally. We presented the letters in a 3 × 3 grid with a central target letter and irrelevant, surrounding letters. We shifted the positions of the letters to hamper processing of the relevant letter and increase processing of the irrelevant letters. First, we varied how far the whole grid was shifted horizontally and vertically from the center of the screen in each trial. We randomly selected a value that was larger than −5, unequal to 0 and smaller than 5 and multiplied it by a random value between 1 and 3. So the shift was between −15 and 15 pixels (without 0) from center.^2^ We computed the shift separately for the horizontal and vertical axes. Second, we shifted each letter randomly at least 1 and up to 5 pixels either to the left or right and to the top or bottom. The space between two letters was 28 pixels on the horizontal axis and 34 pixels on the vertical axis without these shifts.

#### Procedure

The experimenters asked participants about their demographic information. They then placed the keyboard comfortably for responding, depending on the dominant hand of participants. Participants provided informed consent for participation and data protection regulations. Afterward they had to put on the headphones.

Participants read through the instructions on the computer screen at their own pace. The first task was to categorize a letter as fast and as accurately as possible. This task was described as challenging because it required a high pace of responding. Participants were encouraged to improve constantly. Participants had to place their fingers on the response keys and memorize the assignment of letters to these keys, which was presented as text and in a picture. Only the central letter of a grid of letters had to be categorized, and eight additional, surrounding letters had to be ignored. A response to the letter produced a low- or high-pitched sound and the two sounds were played during instructions. The second task was to choose between the three responses freely whenever the low- or high-pitched sound played again. Participants were encouraged not to use conscious strategies for this choice and were asked to respond as fast as possible. Participants were to refrain from responding and wait for the next trial to commence whenever they heard a third sound of medium pitch. This sound was also played during instructions. The instructions closed with a summary of the two parts of a trial, emphasizing that participants should conduct the second part even if they committed an error in the first part. Participants had the chance to go through all instructions again or start the practice block instead.

**Fig. 2:**
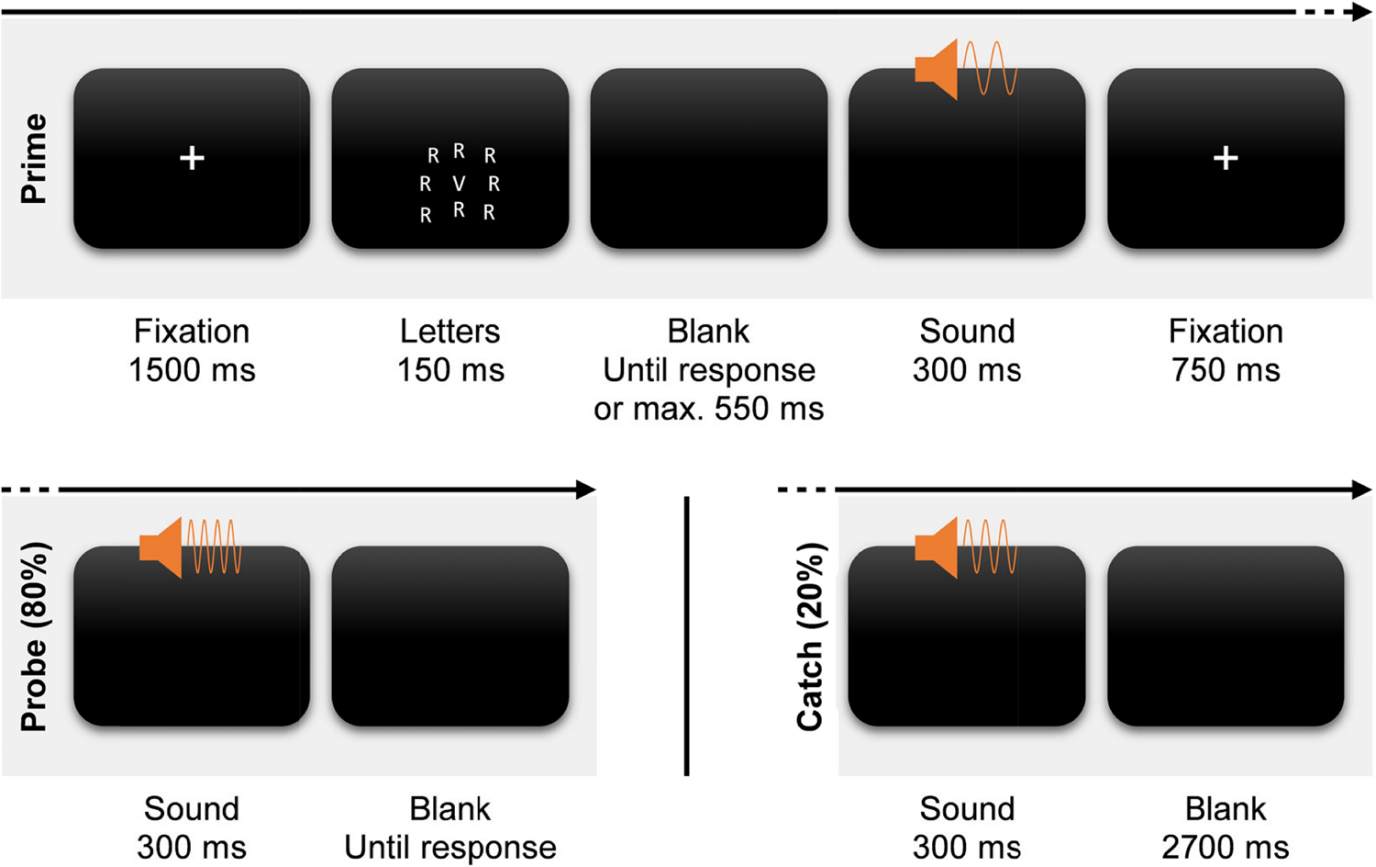
Trial procedure of Experiment 1. *Note.* Each trial started with a prime section (top). After the presentation of a fixation cross for 1,500 ms, nine letters appeared for 150 ms. Participants had to respond to the central letter with one of three response keys within 700 ms from letter onset and ignore the surrounding irrelevant letters. Both letter types were identical in 1/6 of the trials (congruent) and mapped to two different responses in 5/6 of the trials (incongruent). When participants responded, a low- or high-pitched sound played for 300 ms. After another fixation for 750 ms, a low- or high-pitched probe sound played for 300 ms in 80% of the trials (bottom left). These sounds indicated participants to choose one of the three response keys freely and spontaneously. In the other 20% of the trials, a medium-pitched catch sound played, indicating participants to refrain from responding (bottom right)

Each trial started with the prime section (see Fig. 2). A fixation cross appeared for 1500 ms. Then the letter grid was presented and vanished after 150 ms. Participants had 700 ms from letter onset to respond. The response deadline was intentionally short to provoke commission errors. If they responded, the low- or high-pitched sound played for 300 ms. A fixation cross appeared afterward for 750 ms. Then either a probe or catch sound appeared for 300 ms. There was no time limit to deliver a probe response, and the next trial commenced upon responding. In case of catch sounds, participants instead had to refrain from responding and wait for the next trial to commence for 3000 ms from the onset of the catch sound. These catch sounds were implemented to increase attention to the otherwise irrelevant sounds and to reduce preparation of probe responses.

Participants could err throughout the trial and received specific feedback immediately for 2,000 ms for all errors in red font in the first practice block and only for some errors in the following experimental blocks. Errors occurred in the form of responding during fixation before letter onset (timing error), pressing one of the instructed keys but a wrong one for the letters (commission error) or releasing one of these keys before providing a key press to the letter (i.e., a key press started before letter onset and ended during the letter task; release error^3^), not responding to the letters (omission error), responding during the sound or fixation in the prime before the probe (timing error), responding to catch sounds (catch errors) or using any other than the instructed keys for the prime or probe response (random error). In practice trials, we also fed back correct prime, probe and catch responses in green font. In experimental blocks, commission and release errors were not fed back immediately. At the end of each block, the number of correct trials and the average response time in these trials were fed back. An illustration reminded the participants of the assignment of the letters to the response keys. If participants’ accuracy dropped below 50% for prime, probe or catch response or if they preferred (>53%) or avoided (<13%) at least one of the response keys in the probe, they received feedback to improve this/these aspect(s) of the task. If participants received such criterion-based feedback, the experimenter had to press a secret key for participants to proceed to the next block so that they had the opportunity to instruct participants again about the part(s) of the task they struggled with. If they did not receive any criterion-based feedback, they could proceed to the next block by themselves. We implemented adaptive feedback to deter participants from erring too frequently and from biased probe responding.

The first block was considered practice and had 30 trials. The following twelve blocks had 60 trials each. In these experimental blocks, one sixth of the trials were congruent, the others were incongruent. Whereas in congruent trials, relevant and irrelevant letters were the same, they were different and mapped to different responses in incongruent trials. We implemented congruent trials to draw attention to the irrelevant letters and therefore provoke more errors in incongruent trials. The two sounds (400 and 800 Hz) appeared equally often in congruent and incongruent primes, respectively. Each of these sounds was followed by a catch sound in one fifth of the trials, and equally often the sound repeated or changed between prime and probe in the remaining trials. The relevant letter was selected randomly with the constraints to not be the same as the relevant or irrelevant letter from the preceding trial. For incongruent primes, the distractor was selected randomly with the constraints to not map to the same response as the current relevant letter and to not be the same as the preceding relevant or irrelevant letter. We excluded letter repetitions by design to prevent retrieval of responses from a preceding trial via bindings with letter identities.

The participants were debriefed at the end of the experiment. They received partial course credit or monetary compensation for participation.

#### Software

We analyzed the data in R (Version 4.4.1; R Core Team, 2023) and we used the R packages *schoRsch* (Version 1.10; Pfister & Janczyk, 2016), *tidyverse* (Version 2.0.0; Wickham et al., 2019), and *ez* (Version 4.4-0.4; Lawrence, 2016). We implemented a version-controlled analysis by applying these packages as available on June 1, 2024, via the R package *groundhog* (Version 3.2.0; Simonsohn & Gruson, 2024).

#### Data treatment

We excluded the practice block. We computed accuracy separately for prime, catch and probe responses. Fifteen participants had an accuracy below 50% in at least one of the three response types and were excluded and replaced. We excluded catch trials (20%). Afterward, we computed the preference for each response key in the probe. Six participants chose at least one of the three keys in more than 53% or less than 13% of the trials (i.e., more than 20% difference to chance level) and were therefore excluded and replaced. We then selected trials with an incongruent prime (16.7% excluded). We further selected trials with a prime response that was correct, a flanker or a nonflanker commission error (14.6% excluded because of an omission, random, release, or timing error during the sound or fixation between prime and probe). We excluded trials with a random error in the probe (<0.1%).

We then computed how many probe key presses participants delivered for each of the six combinations of sound sequence (repetition vs. change) × prime accuracy (correct vs. flanker error vs. nonflanker error). We had originally preregistered to exclude participants who delivered fewer than 10 observations in any of these cells. After we had collected the fourth pilot sample, we updated the preregistration and lowered this criterion to 5 observations and continued data collection for this fourth version. All remaining participants delivered at least 5 observations in each experimental cell and could be included in the analysis.

### Results

We report detailed descriptive statistics for each analysis in Tables 1, 2, 3 and 4 in Appendix B.

#### Exploratory analysis of the frequency of error types in the prime

The prime responses were 80.5% correct, 10.8% flanker errors and 8.6% nonflanker errors. In other words, 55.7% of the erroneous prime responses mapped to the flanker. An exploratory two-tailed paired-samples *t*-test showed that the frequency of flanker errors was significantly higher than of nonflanker errors, *t*(77) = 5.32, *p* < .001, *d*_*z*_ = 0.60.

#### Main analyses of the response repetition frequency in the probe

**Fig. 3:**
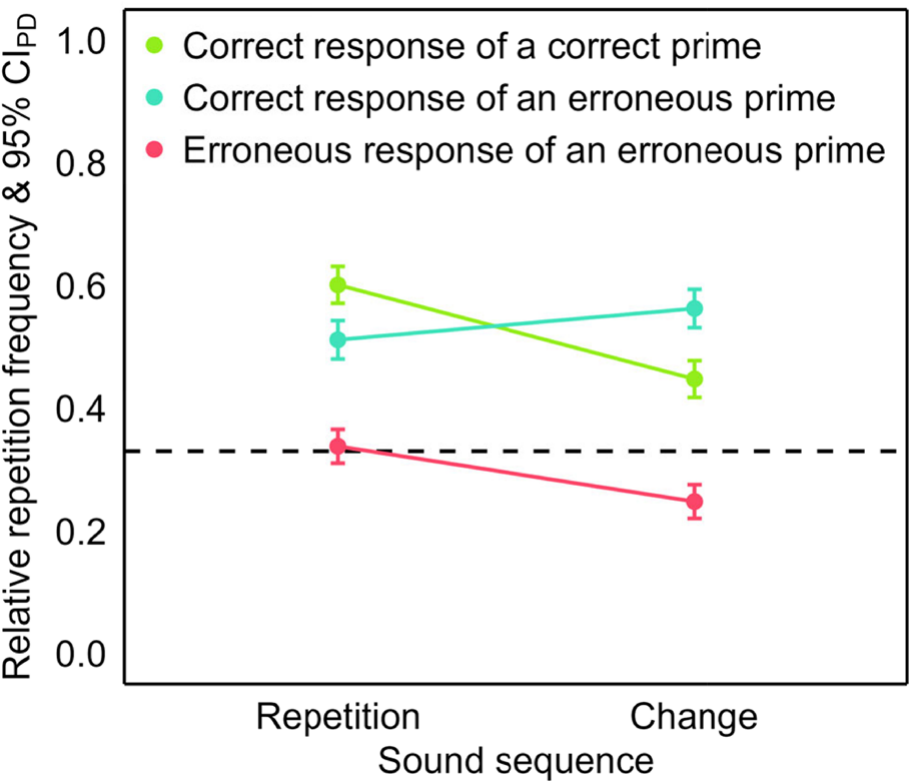
Repetition frequency after correct and erroneous primes in Experiment 1. *Note.* Mean relative repetition frequency of the prime response in the probe as a function of sound sequence (repetition and change) and repeated response (green: correct response of correct prime, teal: correct response of an erroneous prime and pink: erroneous response of an erroneous prime). The error bars are 95% confidence intervals of the paired differences (CI_PD_), visualizing two-tailed, paired-samples *t-*tests between sound sequences. The dashed line visualizes the chance level of response repetitions. (Color figure online)

**Fig. 4:**
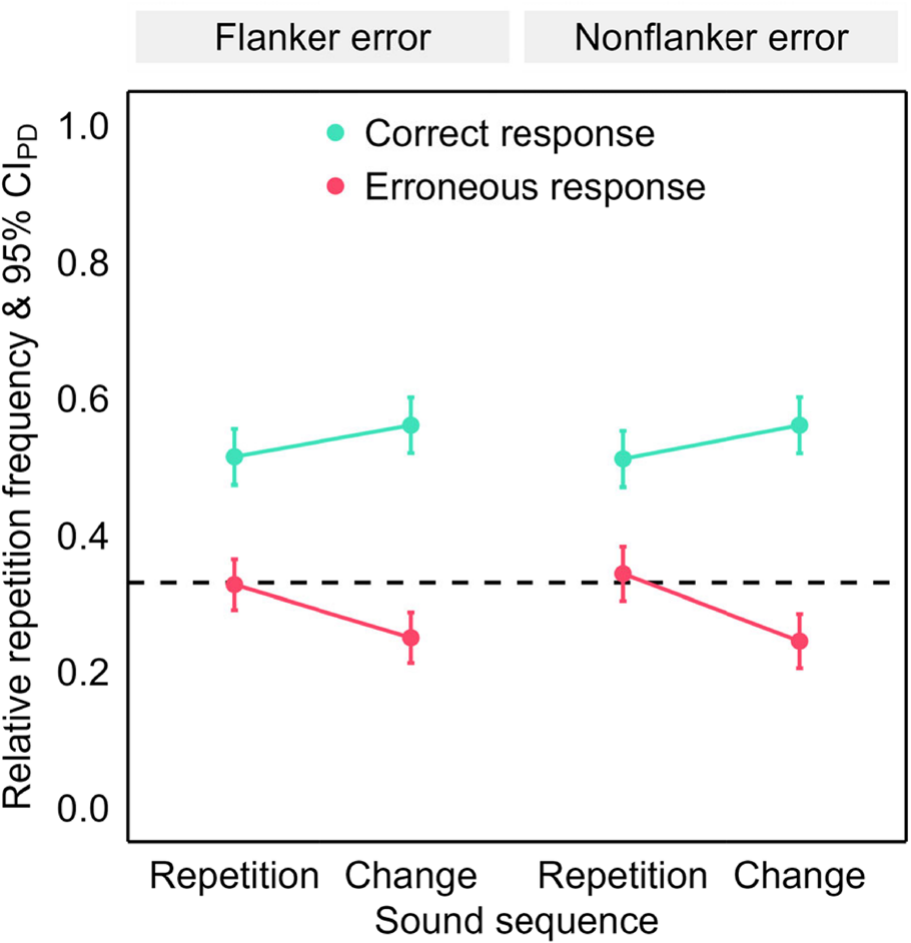
Repetition frequency after erroneous flanker and nonflanker primes in Experiment 1. *Note.* Mean relative repetition frequency of the prime response in the probe as a function of error type (flanker and nonflanker error), sound sequence (repetition and change) and repeated response (teal: correct response and pink: erroneous response). The error bars are 95% confidence intervals of the paired differences (CI_PD_), visualizing two-tailed, paired-samples *t*-tests between sound sequences. The dashed line visualizes the chance level of response repetitions. (Color figure online)

The first analysis in this section compares the impact of sound sequence on repeating correct responses of correct or erroneous primes and erroneous responses of erroneous primes in the probe, without differentiating between flanker and nonflanker errors (see Fig. 3). The second analysis then compares the sound sequence effect for repeating correct and erroneous prime responses between flanker and nonflanker errors in the probe (see Fig. 4). Figure 8 in Appendix C provides an overview of individual sound sequence effects for all repeated responses after prime responses that were correct, flanker errors and nonflanker errors.

We analyzed the repetition frequency^4^ in the probe in an analysis of variance (ANOVA) as a function of the within-subjects factors sound sequence (repetition vs. change) × repeated response (correct response of a correct prime vs. correct response of an erroneous prime vs. erroneous response of an erroneous prime). The main effect of repeated response was significant, *F*(2, 154) = 23.07, *p* < .001, η_p_^2^ = .23 (Greenhouse–Geisser corrected; ε = .84), and the repetition frequency was higher for sound repetitions than changes, *F*(1, 77) = 88.87, *p* < .001, η_p_^2^ = .54. The two factors interacted significantly, *F*(2, 154) = 41.70, *p* < .001, η_p_^2^ = .35 (Greenhouse–Geisser corrected; ε = .73).

We scrutinized the significant main effect of repeated response in separate two-tailed paired-samples *t*-tests, comparing the three types of repeated responses with each other. Both the correct response of a correct prime, *t*(77) = 7.57, *p* < .001, *d*_*z*_ = 0.86, and of an erroneous prime, *t*(77) = 5.32, *p* < .001, *d*_*z*_ = 0.60, were repeated more frequently than the erroneous response of an erroneous prime. The repetition frequency did not differ between both correct responses, |*t*| < 1.

We further scrutinized the significant two-way interaction via comparisons of sound repetitions and changes separately for the three types of repeated responses in two-tailed paired-samples *t*-tests. The repetition frequencies of the correct response of a correct prime, *t*(77) = −10.17, *p* < .001, *d*_*z*_ = −1.15, and of the erroneous response of an erroneous prime, *t*(77) = −6.52, *p* < .001, *d*_*z*_ = −0.74, were higher for sound repetitions than changes. For the correct response of an erroneous prime, the repetition frequency was lower for sound repetitions than changes, *t*(77) = 3.25, *p* = .002, *d*_*z*_ = 0.37. We then compared sound sequence effects between the three types of repeated responses in two-tailed paired-samples *t*-tests. The sound sequence effect was more negative for the correct response of a correct prime than for both the correct response, *t*(77) = −8.60, *p* < .001, *d*_*z*_ = −0.97, and the erroneous response of an erroneous prime, *t*(77) = −4.13, *p* < .001, *d*_*z*_ = −0.47. The correct response of an erroneous prime had a more positive effect than the erroneous response of an erroneous prime, *t*(77) = 5.06, *p* < .001, *d*_*z*_ = 0.57.

We analyzed the repetition frequency in the probe in an ANOVA with the within-subjects factors error type (flanker vs. nonflanker) × sound sequence (repetition vs. change) × repeated response (correct response vs. erroneous response). Mirroring the preceding analysis, we found higher repetition frequencies for the correct than the erroneous response, *F*(1, 77) = 30.04, *p* < .001, η_p_^2^ = .28, as well as for sound repetitions than changes, *F*(1, 77) = 15.08, *p* < .001, η_p_^2^ = .16. These two factors interacted significantly, *F*(1, 77) = 23.64, *p* < .001, η_p_^2^ = .23. The main effect of error type and interactions with this factor were not significant, *F*s < 1.

We scrutinized the significant two-way interaction in two-tailed paired-samples *t-*tests. The repetition frequency was higher for sound repetitions than changes for the erroneous response, *t*(77) = −6.20, *p* < .001, *d*_*z*_ = −0.70, whereas the opposite sound sequence effect emerged for the correct response, *t*(77) = 3.08, *p* = .003, *d*_*z*_ = 0.35.

#### Secondary analysis of the response time in the probe

We analyzed response times in the probe in an ANOVA with the within-subjects factor prime accuracy (correct vs. flanker error vs. nonflanker error). We did not include the factor sound sequence as described for the main analyses because response times are typically noisy in comparable free-choice designs. The main effect of prime accuracy was significant, *F*(2, 154) = 8.65, *p* < .001, η_p_^2^ = .10 (Greenhouse–Geisser corrected; ε = .90). We scrutinized differences between the three prime accuracies in two-tailed, paired-samples *t*-tests. Responding in the probe was faster when the prime was correct (*M* = 655 ms) than when it was a flanker error (*M* = 782 ms), *t*(77) = −4.07, *p* < .001, *d*_*z*_ = −0.46, or a nonflanker error (*M* = 779 ms), *t*(77) = −3.09, *p* = .003, *d*_*z*_ = −0.35. Response times after flanker and nonflanker errors did not differ significantly, |*t*| < 1.

### Discussion

The aim of Experiment 1 was to replicate and generalize binding and retrieval for erroneous responses and their irrelevant effects. Therefore, participants conducted a flanker task in the prime segment of each trial. Each correct response, flanker error and nonflanker error triggered one of two effect sounds. We manipulated whether the same or the other sound appeared afterward in the probe segment of the trial as a signal to choose one of the responses spontaneously. We hypothesized that the repetition of the sound from prime to probe would bias probe responses toward the executed prime response and that the strength of this impact of the sound might differ between flanker and nonflanker errors.

In line with the first part of our hypothesis, repetitions of the executed erroneous prime response in the probe were more frequent when the sound repeated than changed. The results therefore replicate previous findings, corroborating binding and retrieval of the erroneous response and the effect (Exp. 2 in Foerster et al., 2022a). We further provide novel insight with the observation that repetitions of the not executed correct response of an error were less frequent for sound repetitions than changes. This result therefore contradicts the assumption of additional binding between the correct response and the sound. As such, more frequent repetitions of the erroneous response for sound repetitions led to fewer repetitions of the correct response.

At the same time, participants showed a huge general preference for choosing probe responses that matched the not executed correct response instead of the executed erroneous response from an erroneous prime. Further, the frequency of choosing the correct response did not differ between trials with a correct or an erroneous prime. That is, a correct response, independent of whether it was executed, had a huge impact on following action control. After erroneous primes, the correct response was chosen in 53.7%, the erroneous response in 29.3% and the remaining neutral response in only 17.0% of the probes. That is, the erroneous response was preferred over the neutral response. These results corroborate recent findings of increased activity of both the correct and erroneous response after error commission (Foerster et al., in press).

Binding and retrieval effects emerged for both error types but did not differ between them. Differences in binding and retrieval effects might be absent because flanker and nonflanker errors did not differ in the way we assumed they would. First, we assumed less correct response activation for flanker errors where more attention should be on the irrelevant stimuli (Maier et al., 2008). Contradicting this assumption, repetitions of the correct response did not occur more frequently after nonflanker than flanker errors. We randomly shifted the positions of the letters from the screen center. This shift might have hampered correct response activation for nonflanker errors, masking a potential attention deficit for flanker errors. However, the finding of a general preference for the selection of the correct response—independent of whether the prime was correct, a flanker error, or a nonflanker error—contradicts this interpretation. Alternatively, deriving the correct response from the target was probably easier in our study because we assigned only six stimuli to three responses whereas Maier et al. (2008) assigned eight stimuli to four responses. Still, exploratory analyses showed that flanker errors occurred more frequently than nonflanker errors in the current study, indicating that a selection failure in favor of irrelevant stimuli provoked these errors to some extent. Second, we assumed a shift toward more selectivity after flanker than nonflanker errors (Maier et al., 2011), however, we could not test this assumption in our design. In a nutshell, we generalized binding between erroneous responses and effects to a new error type (i.e., flanker errors). However, future research could specify processing differences between error types via comprehensive manipulation checks and their impact on binding and retrieval at the same time.

We also compared binding and retrieval effects after errors and after correct responses. For correct prime responses, sound repetitions promoted repetitions of this response in the probe. This result is in line with similar binding and retrieval effects in previous research with two-choice designs (e.g., Dutzi & Hommel, 2009; Hommel, 1998; Moeller et al., 2016, 2019). In the current study, sound sequence effects were stronger after a correct prime than after an erroneous prime. In the previous study, we only found descriptive differences in binding and retrieval strength, however, the sample size was considerably smaller (Exp. 2 in Foerster et al., 2022a). The analyses of the response times showed a typical slowing in the probe following both types of errors in the prime (e.g., Rabbitt, 1966). A variety of processes have been suggested as a source of this slowing, that is, monitoring (e.g., Jentzsch & Dudschig, 2009), orienting (e.g., Notebaert et al., 2009) or a shift toward more conservative responding (e.g., Laming, 1979). All of these processes potentially hamper binding or retrieval for errors compared with correct responses.

## Experiment 2

Experiment 1 and previous research provide converging evidence for binding between erroneous responses and their effects (Exp. 2 of Foerster, Moeller, et al., 2022a, 2022b). There are also empirical data for binding between irrelevant stimuli and not executed correct responses (Foerster et al., 2021). Participants responded to one out of six letters that were assigned two responses. Importantly, an irrelevant blue or yellow rectangle was presented behind the target letter. When the same color appeared in two consecutive trials, responding in the following trial was faster and more accurate when it matched the correct response from the preceding trial, relative to when the color changed. Such evidence for binding and retrieval of a correct response emerged both when the response in the binding instance was correct and when it was erroneous. Further, the data from a paradigm with three alternative responses suggests that there is only binding of stimulus features with correct response features and no additional, weaker binding with erroneous response features (Parmar et al., 2022).

We propose that response execution serves as an anchor for binding either correct or erroneous response features. That is, irrelevant perceptual features appearing before erroneous response execution should enter bindings with the not executed correct response. In contrast, the same features should bind to the executed erroneous response if they are presented after responding. There is some evidence for independent bindings of irrelevant stimuli and effects with *correctly* executed responses (Moeller et al., 2019). Crucially, bindings did not emerge between all three features or between stimulus and effect features, encouraging our assumption that perceptual features are bound to different response features, depending on their timing.

So far, these two bindings have been studied separately for erroneous responses. The current study will therefore address both bindings 1) in the same experiment and 2) manipulate the same perceptual features for stimuli and effects. We used a similar prime–probe paradigm as in Experiment 1. Crucially, the sound in the prime was presented as a stimulus, that is, before response execution, or as an effect, that is, after response execution. Again, binding of an executed (correct or erroneous) or not executed correct response to the sound in the prime would be reflected in increased response repetition frequencies of the respective response for sound repetitions compared to changes. We expected sound repetitions relative to changes to increase the repetition frequency for the correct response of a correct prime in the probe. This increase should emerge when the sound appears before and after correct response execution in the prime. For erroneous primes, we expected sound repetitions relative to changes to increase the repetition frequency 1) for the erroneous response when prime sounds appear after response execution and 2) for the correct response when prime sounds appear before response execution. The comparison of these binding effects between flanker and nonflanker errors was considered secondary in this experiment because we expected fewer participants to provide sufficiently many observations with the addition of the factor prime sound timing.

### Method

#### Participants

In Experiment 1, differences between sound changes and sound repetitions, indicative of binding between an executed response and the following irrelevant effect, were |*d*_*z*_| ≥ 0.37 for the three repeated responses (correct response of a correct prime, correct response of an erroneous prime and erroneous response of an erroneous prime). Pairwise comparisons of the sound sequence effects between the three response choices amounted to |*d*_*z*_| ≥ 0.47. Effects indicative of binding between an irrelevant stimulus and the correct response in response times were *d*_*z*_ = 0.40 for erroneous responses and *d*_*z*_ = 0.48 for correct responses in a preceding study of us (Foerster et al., 2021).

A sample of 60 participants has a power of 80% to detect the smallest effect size (*d*_*z*_ = 0.37) in a two-tailed test with α = 5% (computed with the *power.t.test* function in R Version 4.3.1). We collected data until we had 60 analyzable datasets. As in Experiment 1, we planned to collect pilot samples of up to eight participants each. The first pilot was successful, so we filled up the sample. One participant changed their hand for responding in the middle of the experiment and we decided to exclude this dataset because of this deviation from the experimental procedure. For two participants, the computer crashed during the experiment and five participants aborted the study prematurely. We replaced these partial datasets. Ninety-two participants provided full datasets of which we had to exclude 32 participants as per our preregistered criteria. Of the remaining 60 participants, 41 reported themselves to be female, 18 male and one nonbinary; 54 right-handed, four left-handed, two ambidextrous. The mean age was 26.8 years (*SD* = 6.40 years).

#### Procedure

The apparatus was the same as in Experiment 1 and we only made a few changes in the procedure. We only address these changes in the following for brevity.

After learning about the relevant and irrelevant letters, participants read in the instructions that a low- or high-pitched sound could be played. The sound would either precede their key press or would be produced by their key press.

**Fig. 5:**
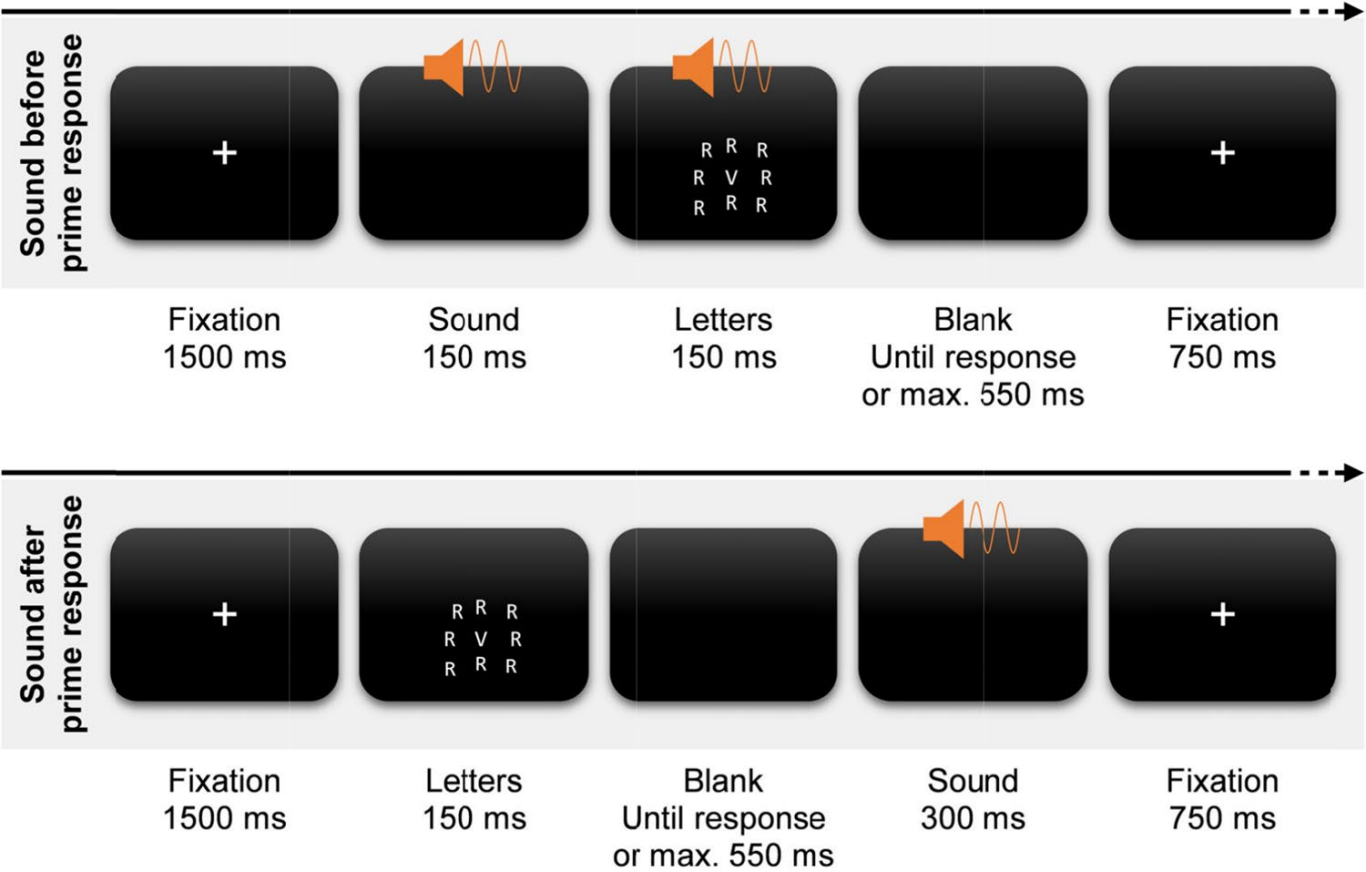
Trial procedure of Experiment 2. *Note.* Each trial started with a prime section. Half of the trials featured the same prime procedure as in Experiment 1 with the sound playing after participants delivered a prime response (bottom). The other half of the trials instead presented the sound before the prime response for 150 ms before letter onset and during letter presentation. The catch and probe procedure were identical to Experiment 1 (see Fig. 2)

We adapted the prime section of the trial (see Fig. 5). We manipulated whether the low- or high-pitched sound appeared before or after the prime response. In one half of the trials, the sound was played for 150 ms before letter onset during a blank screen and for another 150 ms during letter presentation. In the other half of the trials, the sound played after responding during a blank screen for 300 ms. Notably, the prime ended with the onset of the prime response when the sound played before, but it ended with the sound when this sound played after the prime response. As such, the interval between the occurrence of the last element of the assumed binding (response vs. sound) and the probe sound was comparable.

For the 12 experimental blocks à 60 trials, we presented a random sequence of 96 trials with probe sounds and 24 trials with catch sounds across two blocks. The trials with probe sounds featured each combination of 2 prime sound timing × 2 prime sound pitch × 2 sound sequence twice with congruent primes and ten times with incongruent primes. The trials with catch sounds featured each combination of 2 prime sound timing × 2 prime sound pitch once with congruent primes and five times with incongruent primes.

#### Software

We used the same analysis tools as in Experiment 1. We additionally used the R package *ggh4x* (Version 0.2.8; van den Brand, 2024).

#### Data treatment

We excluded the practice block. We then computed the mean accuracy for each participant, separately for prime, catch and probe responses. Primes were incorrect when participants responded early during fixation or sound presentation before the prime letters appeared, when they omitted a response or delivered a wrong response to the prime letters and when they delivered multiple key presses between prime letter and probe sound onset. Responding to catch sounds was incorrect. Probes were incorrect when participants pressed any other than the three instructed keys. We excluded and replaced 23 participants for whom the accuracy of any of the three values was below 50%. We then excluded catch trials (20%). Afterward we computed the preference for each response key in the probe. We excluded and replaced one participant who chose at least one of the three keys in more than 53% or less than 13% of the trials. We then selected trials with an incongruent prime (16.7% excluded) and with a correct response or a commission error in the prime (14.5% other prime errors excluded). We further excluded trials with a random key press in the probe (< 0.1%). Finally, we excluded all trials with a key press during the presentation of the letters in the prime (response time ≤ 50 ms; 0.1%) because these fast responses coincide with the sound playing in trials with an early sound timing. Eight participants delivered less than 5 observations in at least one of the eight combinations of sound timing (before vs. after prime response), sequence of irrelevant sounds (repetition vs. change) × prime response (correct vs. commission error). We excluded and replaced these participants.

### Results

We report detailed descriptive statistics for each analysis in Tables 5, 6, 7 and 8 in Appendix B.

#### Exploratory analysis of the frequency of error types in the prime

The prime responses were 80.4% correct, 11.1% flanker errors and 8.5% nonflanker errors. In other words, 56.6% of the erroneous prime responses mapped to the flanker. An exploratory two-tailed paired-samples *t*-test showed that the frequency of flanker errors was significantly higher than of nonflanker errors, *t*(59) = 8.10, *p* < .001, *d*_*z*_ = 1.05.

#### Main analysis of the response repetition frequency in the probe

**Fig. 6:**
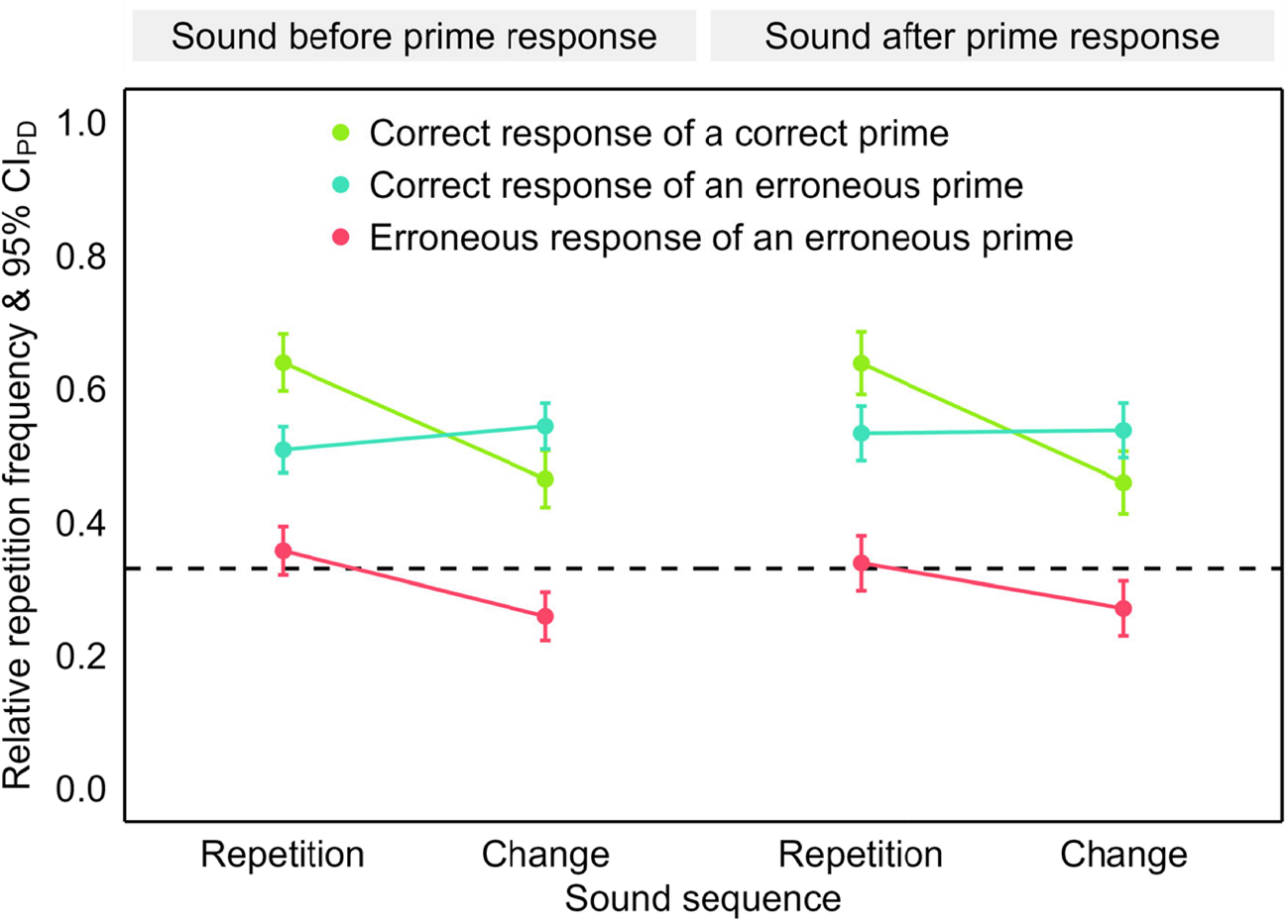
Repetition frequency after correct and erroneous primes in Experiment 2. *Note.* Mean relative repetition frequency of the prime response in the probe as a function of sound timing (before and after prime response), sound sequence (repetition and change) and repeated response (green: correct response of correct prime, teal: correct response of an erroneous prime and pink: erroneous response of an erroneous prime). The error bars are 95% confidence intervals of the paired differences (CI_PD_), visualizing two-tailed, paired-samples *t*-tests between sound sequences. The dashed line visualizes the chance level of response repetitions. (Color figure online)

We analyzed the repetition frequency in the probe in an ANOVA (see Fig. 6 and Fig. 9) as a function of the within-subjects factors sound timing (before vs. after prime response) × sound sequence (repetition vs. change) × repeated response (correct response of a correct prime vs. correct response of an erroneous prime vs. erroneous response of an erroneous prime). The main effect of repeated response was significant, *F*(2, 118) = 12.87, *p* < .001, η_p_^2^ = .18 (Greenhouse–Geisser corrected; ε = .73), and the repetition frequency was higher for sound repetitions than changes, *F*(1, 59) = 77.68, *p* < .001, η_p_^2^ = .57. The two factors interacted significantly, *F*(2, 118) = 31.16, *p* < .001, η_p_^2^ = .35. The main effect of sound timing as well as the two-way interactions including this factor were not significant, *F* < 1. The three-way interaction was also not significant, *F*(2, 118) = 1.54, *p* = .223, η_p_^2^ = .03 (Greenhouse–Geisser corrected; ε = .66).

We compared the three types of repeated responses in separate two-tailed paired-samples *t*-tests to scrutinize the significant main effect of repeated response. Both the correct response of a correct prime, *t*(59) = 7.20, *p* < .001, *d*_*z*_ = 0.93, and of an erroneous prime, *t*(59) = 3.56, *p* = .001, *d*_*z*_ = 0.46, were repeated more frequently than the erroneous response of an erroneous prime. The repetition frequency did not differ between both correct responses, |*t*| < 1.

We then scrutinized the significant two-way interaction between sound sequence and repeated response. First, we compared sound repetitions and changes separately for the three types of repeated responses in two-tailed paired-samples *t*-tests. The repetition frequencies were higher for sound repetitions than changes for the correct response of a correct prime, *t*(59) = −8.28, *p* <.001, *d*_*z*_ = −1.07, and for the erroneous response of an erroneous prime, *t*(59) = −5.61, *p* < .001, *d*_*z*_ = −0.72. For the correct response of an erroneous prime, the sound sequence effect was not significant, *t*(59) = 1.43, *p* = .158, *d*_*z*_ = 0.18. Second, we compared the sound sequence effects between the three types of repeated responses in two-tailed paired-samples *t*-tests. The sound sequence effect was more negative for the correct response of a correct prime than for the correct response, *t*(59) = −7.49, *p* < .001, *d*_*z*_ = −0.97, and the erroneous response of an erroneous prime, *t*(59) = −4.30,* p* < .001, *d*_*z*_ = −0.55. The correct response of an erroneous prime had a more positive effect than the erroneous response of an erroneous prime, *t*(59) = 3.89, *p* < .001, *d*_*z*_ = 0.50.

#### Secondary analysis of the response repetition frequency in the probe

For this secondary analysis, we additionally excluded 31 participants who did not provide at least five observations in each of the eight combinations of sound timing (before vs. after prime response) × sound sequence (repetition vs. change) × error type (flanker vs. nonflanker). We expected fewer participants than for the main analysis and we did not replace excluded participants. The erroneous prime responses were 55.0% flanker errors.

**Fig. 7:**
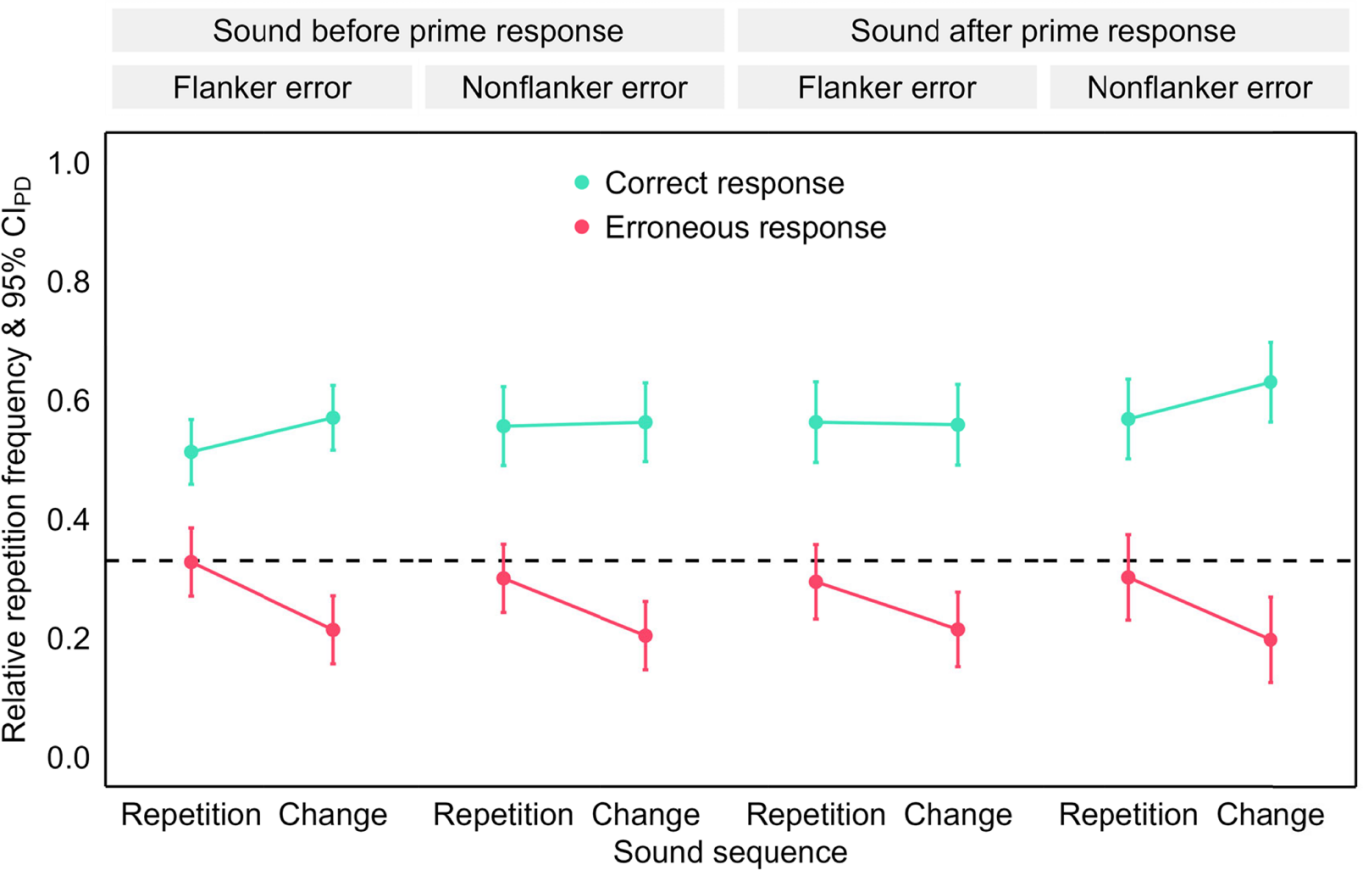
Repetition frequency for erroneous flanker and nonflanker primes in Experiment 2. *Note.* Mean relative repetition frequency of the prime response in the probe as a function of sound timing (before and after prime response), error type (flanker and nonflanker error), sound sequence (repetition and change) and repeated response (teal: correct response and pink: erroneous response). The error bars are 95% confidence intervals of the paired differences (CI_PD_), visualizing two-tailed, paired-samples *t*-tests between sound sequences. The dashed line visualizes the chance level of response repetitions. (Color figure online)

**Fig. 8:**
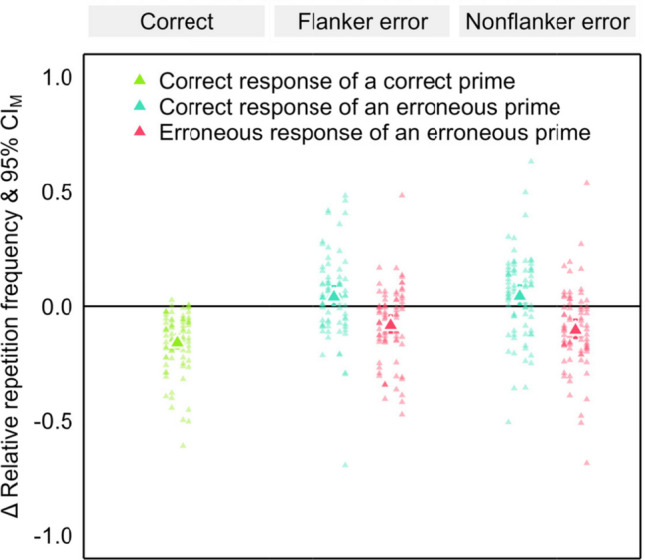
Descriptive statistics for sound sequence effects in Experiment 1. *Note.* Mean difference between the relative repetition frequency of sound changes and repetitions (Δ) for each participant (transparent, small triangles) and cell (opaque, large triangles) as a function of prime accuracy (correct, flanker error and nonflanker error) and repeated response (green: correct response of correct prime, teal: correct response of an erroneous prime and pink: erroneous response of an erroneous prime). The error bars are 95% confidence intervals of the means (CI_M_), visualizing two-tailed one-sample *t*-tests of the sound sequence effects against 0. (Color figure online)

**Fig. 9:**
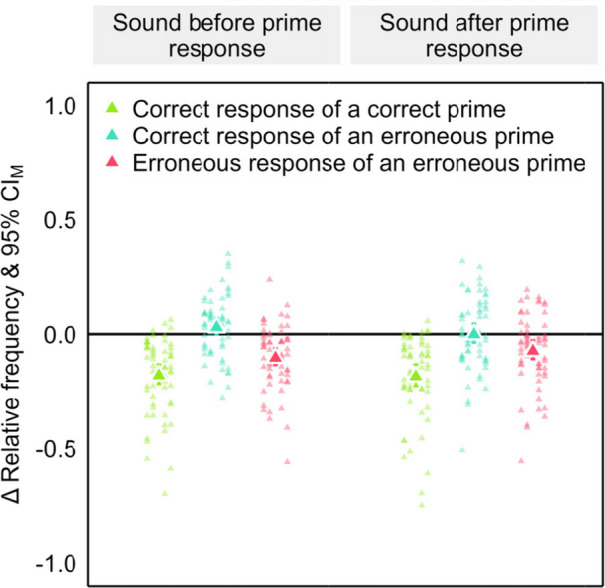
Descriptive statistics for sound sequence effects in Experiment 2. *Note.* Mean difference between the relative repetition frequency of sound changes and repetitions (Δ) for each participant (transparent, small triangles) and cell (opaque, large triangles) as a function of sound timing (before vs. after prime response) and repeated response (green: correct response of correct prime, teal: correct response of an erroneous prime and pink: erroneous response of an erroneous prime). The error bars are 95% confidence intervals of the means (CI_M_), visualizing two-tailed one-sample *t*-tests of the sound sequence effects against 0. (Color figure online)

We analyzed the repetition frequency in the probe (see Fig. 7) in an ANOVA with the within-subjects factors sound timing (before vs. after prime response) × sound sequence (repetition vs. change) × repeated response (correct response vs. erroneous response) × error type (flanker vs. nonflanker). In line with the main analysis, repetition frequencies were higher for the correct than the erroneous response, *F*(1, 28) = 16.81, *p* < .001, η_p_^2^ = .38, and for sound repetitions than changes, *F*(1, 28) = 49.92, *p* < .001, η_p_^2^ = .64. These two factors interacted significantly, *F*(1, 28) = 14.80, *p* = .001, η_p_^2^ = .35. The remaining main effects, *F*(1, 28) ≤ 2.31, *p* ≥ .140, η_p_^2^ ≤ .08, two-way interactions, F(1, 28) ≤ 2.49, *p* ≥ .126, η_p_^2^ ≤ .08, all three-way interactions, *F*(1, 28) ≤ 1.45, *p* ≥ .238, η_p_^2^ ≤ .05, and the four-way interaction, *F*(1, 28) = 2.20, *p* =.149, η_p_^2^ =.07, were not significant.

We scrutinized the significant two-way interaction between sound sequence and repeated response in two-tailed paired-samples *t*-tests. The repetition frequency was higher for sound repetitions than changes for the erroneous response, *t*(28) = −5.16, *p* < .001, *d*_*z*_ = −0.96, whereas a nonsignificant opposite sound sequence effect emerged for the correct response, *t*(28) = 1.94, *p* = .062, *d*_*z*_ = 0.36.

#### Secondary analysis of the response time in the probe

We tested whether response times in the probe were higher after erroneous than after correct prime responses in a one-tailed paired-samples *t*-test. Responding in the probe was slower when the prime response was erroneous (*M* = 759 ms) than correct (*M* = 664 ms), *t*(59) = 3.88, *p* < .001, *d*_*z*_ = 0.50.

### Discussion

The aim of Experiment 2 was to explore whether erroneous response execution serves as an anchor for binding so that perceptual features appearing before response execution are bound to the correct response and perceptual features appearing after response execution are bound to the erroneous response. Therefore, participants conducted a flanker task in the prime segment of each trial. An irrelevant sound was played as stimulus or as effect, that is, before or after response execution. We manipulated whether the same or the other sound appeared afterward in the probe segment of the trial as a signal to choose one of the responses spontaneously. We hypothesized that the repetition compared with the change of the sound from prime to probe would bias probe responses toward the executed correct prime response, independent of whether the prime sound appeared as stimulus or effect. For erroneous primes, we hypothesized that repetitions of sound stimuli would bias probe responses toward the not executed correct prime response and that repetitions of sound effects would bias probe responses toward the executed erroneous prime response (both relative to sound changes).

First, we replicated the finding from Experiment 1 of increased repetitions of the executed erroneous prime response in the probe for effect sound repetitions compared to changes. We therefore have ample evidence for binding of erroneous responses and their irrelevant effects (Exp. 2 in Foerster et al., 2022a). In contrast to our hypothesis and to preceding studies (Foerster et al., 2021; Parmar et al., 2022), the data support binding between irrelevant stimuli and erroneous responses because repetitions of sound stimuli increased the repetitions of the executed erroneous prime response in the probe. Analogously to Experiment 1, the data pattern does not support the assumption of additional binding between the not executed correct responses and either sound stimuli or effects. The frequency of repetitions of this response did not differ significantly between sound sequences.

The binding of stimuli to the erroneous response could come about when errors are not detected and there is only weak activation of the correct response. However, we again observed the typical slowing in the probe after an error in the prime, pointing to error processing (e.g., Rabbitt, 1966). Such error processing might be at the heart of reduced sound effects after erroneous primes compared to after correct primes in both experiments. Further, we also found similar high preferences for choosing the correct response after correct and erroneous primes, and participants again chose the erroneous response more frequently than the neutral response after erroneous primes. We therefore accumulated converging evidence for increased activity of correct and erroneous responses after erroneous actions (see also Foerster et al., in press). Although the correct response therefore seemed to be available in principle, it did not enter bindings with the sound.

Alternatively, qualitatively different action plans might have led to either binding of irrelevant stimuli to the correct response in previous research or to the erroneous response here. In particular, errors might have emerged because of strong, spontaneous activations of a wrong motor pattern despite an already established correct action plan in previous studies (Foerster et al., 2021; Parmar et al., 2022). In the current paradigm, we introduced additional irrelevant stimuli that mapped to responses and that had to be ignored. Especially in the case of flanker errors, action plans might have therefore been wrong in the first place. The correct response might have only been activated afterward, leading to the observed preferences for it in the probe. In line with these assumptions, relevant and irrelevant stimulus features could have been bound to the correct response in previous paradigms but to the erroneous response in the current paradigm during action planning. Although established paradigms exist to investigate bindings for action plans (e.g., Mocke et al., 2020; Stoet & Hommel, 1999), differentiating correct and erroneous action plans is not straightforward.

Another alternative account holds that binding for the not executed correct response does not emerge right at the moment of error commission, but rather during action monitoring and evaluation. That is, following conscious error detection, human agents might mentally simulate an instance of correctly responding to the preceding target stimulus so that bindings for the erroneous response are assembled online during action performance whereas bindings for the correct response are assembled only offline later. Previous studies have shown that mental simulation is indeed sufficient to craft bindings between stimuli and responses (Cochrane & Milliken, 2019). Whether and how such simulation processes take place likely depends on situational characteristics such as the available time for engaging in simulation, and the complexity of the relevant stimuli. Subtle differences between the present setup and previous work (Foerster et al., 2021; Parmar et al., 2022) might thus have allowed for simulation to occur in previous setups but not in the present one.

There were a few other methodological differences between the current and the two former studies that investigated binding for irrelevant stimuli (Foerster et al., 2021; Parmar et al., 2022). We investigated binding here for sounds with a forced-choice prime and a free-choice probe, while the other studies used colors or sounds in a complete forced-choice design. Whereas Foerster et al. (2021) assigned six target stimuli to two responses, Parmar et al. (2022) assigned three target stimuli to three responses, we assigned six target stimuli to three responses. It is not obvious though how these methodological differences should modulate whether the correct or the erroneous response is bound to the irrelevant stimulus.

The manipulation of sound timing introduced differences in the procedures of these conditions (see Fig. 5). Crucially, an additional event occurred between the prime response and the onset of the probe when the sound played after the prime response but not when it played before the response. As such, the interval between the prime response and the probe sound was longer when the sound played after the prime response. We accepted this difference because we prioritized similar intervals between the occurrence of the last element of the assumed binding and the probe sound across both sound timings. That is why we presented the fixation of the probe immediately after 1) the response when the sound played before responding and 2) the sound that played after the prime response. We assumed that decay of the bindings should therefore be similar for both sound timings. At the same time, this design choice introduced less time for correct response activation in the condition without an effect sound. However, the frequency of selecting the correct response from the prime in the probe was not modulated by sound timing. The absence of such a modulation suggests that the difference in duration of 300 ms before probe onset did not affect correct response activation. Previous research assessed the time between the initiation of an overt correction response after the execution of an erroneous response (e.g., Fiehler et al., 2005; Rabbitt, 2002). These correction response times were much shorter than the duration of the fixation that we presented before the onset of the probe sound. Taken together, it seems unlikely that correct response activation was affected by the duration of the interval between the erroneous prime response and the onset of the probe sound.

In line with Experiment 1, we did not find any modulatory impact of error type on choices in the probe. However, as expected, this secondary analysis had considerably less statistical power because of the largely reduced sample size.

## General discussion

The first aim of this research was to replicate and generalize binding and retrieval for erroneous responses and their irrelevant effects and the second aim was to scrutinize whether perceptual features bind to correct or erroneous responses depending on whether they appear before or after response execution. Regarding the first aim, two experiments provided corroborating evidence for binding and retrieval between erroneous responses and the effects following these responses (Exp. 2 of Foerster et al., 2022a). Further, the data suggested that such binding and retrieval emerge across different types of errors. Regarding the second aim of the study, response execution does not seem to serve as an anchor for binding preceding (i.e., stimuli) or following (i.e., effects) perceptual features with either the correct or the erroneous response, respectively. The evidence instead supports binding with the erroneous response, independent of the timing of perceptual features. This finding is surprising, as previous experiments delivered clear-cut evidence for binding of irrelevant stimuli with correct responses instead (Foerster et al., 2021; Parmar et al., 2022).

Stimulus and effect features might be bound to the same response features whenever response execution aligns with established action plans. This assumption does not imply that all three features are bound into a single compound. In the current study, we manipulated stimulus and effects sound sequences in separate trials. We therefore cannot assess their (in)dependency. At least for correct actions, a previous examination of the sequential effects of all features suggests that bindings between stimuli and responses and between responses and effects are retrieved independently (Moeller et al., 2019). It appears very plausible that retrieval is independent because separate bindings are established at different time points. That is, stimulus–response bindings emerge during action planning and response–effect bindings after response execution when the effect is perceived. At least for bindings between different response features, there is ample evidence that these bindings are already established during action planning before the execution of these plans (e.g., Mocke et al., 2020; Stoet & Hommel, 1999).

Recent evidence further suggests that timing matters considerably for binding to emerge between irrelevant perceptual features and response features (He & Pratt, 2025). In this study, irrelevant perceptual features always appeared relatively long after a response had been cued, that is, after a response plan had been established in the prime segment. For half of the participants, these perceptual features appeared before the execution of the prime response and for the other half after. Only if the perceptual features appeared after but not before response execution in the prime, the performance data in the probe pointed to retrieval of the prime response upon repetition of the perceptual features. Speculatively, irrelevant perceptual features enter bindings with response features only if they appear temporally close enough to response planning or execution. That is, instead of assuming response execution alone as an anchor for binding in errors, both response planning and execution might instead be independent instances of binding concurrently active response and perceptual features.

For *relevant* stimuli, previous evidence unanimously points to binding with the correct response even for errors (Exp. 1 in Foerster et al., 2022a; Foerster et al., 2023). In these studies, participants responded to letters with key presses. At least two letters were assigned to each response. Importantly, responding was faster in the trial after an error if the correct response was repeated in the presence of the same relevant letter as in the preceding trial compared to another letter. This finding suggests that a letter repetition retrieved the preceding correct response. We argued that these bindings therefore steer agents toward successful action control in the future because they mirror established response rules. The current results on binding of irrelevant stimulus features instead introduce a viable alternative: Bindings of relevant stimulus and response features could instead reflect transient action plans, no matter whether these were correct or erroneous. Previous studies might have captured errors with predominantly correct action plans as discussed above for irrelevant features. Binding between relevant stimulus features and response features in errors should therefore also be examined in situations that provoke erroneous action plans.

If evidence for retrieval of bindings of the correct response from a relevant stimulus still emerge in situations with wrong action plans, binding and retrieval can truly be interpreted as a corrective force in action control. Alternatively, the pattern could be similar to the one observed here, suggesting that erroneous action plans are bound and retrieved. In other words, binding would then not reflect corrective efforts after the error, but it would rather reveal how the error occurred. Finally, neither the correct nor the erroneous response might be bound and retrieved. In a recent study, participants were forced to guess the unknown correct response to a picture in a prime and received accuracy feedback immediately after guessing (Foerster et al., 2024). In the probe, the former relevant picture could appear again as an irrelevant feature and the response also either repeated or changed. The evidence pointed to traditional binding and retrieval between pictures and responses whenever the guessed prime response was fed back as correct but not when it was fed back as wrong. One possible interpretation of the results is that there was binding during action planning, which remained intact only after receiving correct feedback but was unbound after erroneous feedback. Recent evidence revealed that bindings between response features of an action plan can be unbound when the action plan is discarded before execution (Mocke et al., 2024). Analogously, if wrong action plans are executed but registered as error, unbinding between relevant stimuli and erroneous responses might take place.

## Conclusion

The two experiments deliver corroborating evidence for the involvement of binding and retrieval in different erroneous actions. Both irrelevant stimuli and effects can be bound to erroneous responses. We propose that the diverging findings across studies for binding of irrelevant stimuli in errors can be attributed to qualitive differences in action planning or monitoring.

## Data Availability

See Open Practices Statement.

## Appendix A

### Pilot studies for Experiment 1

The letter grid was presented centrally in pilot 1 to 3. We reduced the number of blocks from 19 to 13 after the first pilot.

The response deadline was 750 ms in Pilot 1 and 700 ms in all three following versions of the experiment. Error feedback was presented for 1,000 ms in Pilot 1, 1,500 ms in Pilot 2 and 3, and 2,000 ms in Pilot 4. We introduced immediate feedback for timing errors during a trial and included an illustration of the assignment of the letters to the response keys at the end of each block starting from Pilot 2 and continuing in all subsequent pilots. All these procedural changes were intended to reduce the occurrence of errors that were not commission errors. For the same reason and for deterring participants from biased responding in the free-choice task, we provided adaptive performance feedback based on three criteria at the end of a block from Pilot 2 and onward (see *Procedure* of Experiment 1 for details). In Pilot 3 and 4, we intended to boost the impact of this feedback. Therefore, only the experimenter was able to start the next block when participants received it.

## Appendix B

**Table 1:** Descriptive statistics for the frequency of erroneous primes in Experiment 1

Prime error type	Trial number	Frequency in the prime	
	*M*	*M* in %	*SE*
Flanker error	48.2	10.8	0.57
Nonflanker error	38.3	8.6	0.42

*M* = mean, *SE* = standard error.

**Table 2:** Descriptive statistics for the repetition frequency in the probe after correct and erroneous primes in Experiment 1

Sound sequence	Prime accuracy	Repeated response	Trial number *M*	Repetition frequency in the probe	
				*M* in %	*SE*
Repetition	Correct	Correct	108.4	60.1	3.50
	Erroneous	Correct	23.2	51.1	2.51
		Erroneous	14.6	33.8	2.80
Change	Correct	Correct	79.2	44.7	3.68
	Erroneous	Correct	24.3	56.2	2.23
		Erroneous	11.1	24.8	2.44

*M* = mean, *SE* = standard error.

**Table 3:** Descriptive statistics for the repetition frequency in the probe after erroneous primes in Experiment 1

Prime error type	Sound sequence	Repeated response	Trial number	Repetition frequency in the probe	
			*M*	*M* in %	*SE*
Flanker	Repetition	Correct	13.5	51.4	2.83
		Erroneous	8.2	32.7	3.07
	Change	Correct	13.4	56.1	2.40
		Erroneous	6.4	25.0	2.55
Nonflanker	Repetition	Correct	10.2	51.1	2.44
		Erroneous	7.0	34.3	2.74
	Change	Correct	13.4	56.1	2.41
		Erroneous	6.4	24.5	2.55

*M* = mean, *SE* = standard error.

**Table 4:** Descriptive statistics for the response time in the probe after correct and erroneous primes in Experiment 1

Prime accuracy	Trial number	Response time in the probe	
	*M*	*M* in ms	*SE*
Correct	357.6	655	37.4
Flanker error	48.2	782	53.4
Nonflanker error	38.3	779	58.2

*M* = mean, *SE* = standard error

**Table 5:** Descriptive statistics for the frequency of erroneous primes in Experiment 2

Prime error type	Trial number	Frequency in the prime	
	*M*	*M* in %	*SE*
Flanker error	45.2	11.1	0.58
Nonflanker error	34.6	8.5	0.53

*M* = mean, *SE* = standard error

**Table 6:** Descriptive statistics for the repetition frequency in the probe after correct and erroneous primes in Experiment 2

Sound timing	Sound sequence	Prime accuracy	Repeated response	Trial number *M*	Repetition frequency in the probe	
					*M* in %	*SE*
Sound before prime response	Repetition	Correct	Correct	52.9	63.9	4.28
		Erroneous	Correct	11.3	50.8	3.44
			Erroneous	8.6	35.7	3.77
	Change	Correct	Correct	39.4	46.4	4.30
		Erroneous	Correct	12.7	54.4	2.86
			Erroneous	6.3	25.9	3.42
Sound after prime response	Repetition	Correct	Correct	51.5	63.8	4.26
		Erroneous	Correct	9.9	53.3	3.32
			Erroneous	6.7	33.8	3.76
	Change	Correct	Correct	37.6	45.9	4.35
		Erroneous	Correct	10.0	53.7	3.51
			Erroneous	5.6	27.1	3.49

*M* = mean, *SE* = standard error

**Table 7:** Descriptive statistics for the repetition frequency in the probe after erroneous primes in Experiment 2

Sound timing	Prime error type	Sound sequence	Repeated response	Trial number *M*	Repetition frequency in the probe	
					*M* in %	*SE*
Sound before prime response	Flanker	Repetition	Correct	6.4	51.3	4.21
			Erroneous	5.5	32.8	4.58
		Change	Correct	7.2	57.1	3.72
			Erroneous	4.4	21.4	4.03
	Nonflanker	Repetition	Correct	5.3	55.6	4.75
			Erroneous	3.9	30.0	5.11
		Change	Correct	5.6	56.3	4.17
			Erroneous	3.2	20.4	4.10
Sound after prime response	Flanker	Repetition	Correct	5.6	56.3	4.43
			Erroneous	4.2	29.5	4.95
		Change	Correct	5.7	55.9	3.68
			Erroneous	3.7	21.4	4.35
	Nonflanker	Repetition	Correct	4.6	56.8	4.48
			Erroneous	3.6	30.2	4.70
		Change	Correct	5.0	63.0	4.76
			Erroneous	2.9	19.7	3.95

*M* = mean, *SE* = standard error

**Table 8:** Descriptive statistics for the response time in the probe after correct and erroneous primes in Experiment 2

Prime accuracy	Trial number *M*	Response time in the probe	
		*M* in ms	*SE*
Correct	326.7	664	35.5
Erroneous	79.9	759	48.4

*M* = mean, *SE* = standard error

## Appendix C

Figures 8 and 9

## Footnotes

1 These are the two bindings that are relevant for the current research question. However, they are only two examples of a variety of bindings in action control (e.g., Benini et al., 2024; Dignath et al., 2019; Schiltenwolf et al., 2024; Whitehead & Egner, 2025).

2 This computation could not produce a uniform distribution of shifts because some values in the range were not possible (e.g., 7), others were possible only for one multiplication factor (e.g., 15) and others could appear for two multiplication factors (e.g., 4). However, the shift should still have fulfilled its intended purpose of provoking distraction from the task.

3 Key presses are usually short so that release errors are rare events. To make sure that we captured binding and retrieval of *one* response event, we identified and excluded trials where another response was still active.

4 Following the suggestion of a reviewer, we arcsine transformed relative repetition frequencies for each participant and cell in Experiments 1 and 2. The analyses of this transformed data replicated the analyses of the untransformed data reported here. The *R* code for these exploratory analyses is also available at the Open Science Framework (https://osf.io/6s8ey).

## Code availability

See Open Practices Statement.

## Declarations

### Open practices statement

For Experiment 1, the preregistration (https://osf.io/q64dz), which we adapted after collecting our fourth pilot sample (see section *Participants* for details), the data (trial-level) and the analysis code are publicly accessible at the Open Science Framework (https://osf.io/6s8ey). We preregistered to investigate two different processes, namely (1) binding and retrieval as well as (2) error cancellation in Experiment 1. Per our preregistration, we only present the results on binding and retrieval here and we will publish our results on error cancellation separately to make the two topics more accessible for readers. For Experiment 2, we also made the preregistration (https://osf.io/u497x), the data (trial-level) and the analysis code publicly available (https://osf.io/r4fm7).
